# Loss of a newly discovered microRNA in Chinese hamster ovary cells leads to upregulation of N‐glycolylneuraminic acid sialylation on monoclonal antibodies

**DOI:** 10.1002/bit.28015

**Published:** 2022-01-14

**Authors:** Simon Fischer, Sven Mathias, Anna Stadermann, Shumin Yang, Valerie Schmieder, Nikolas Zeh, Nicoletta Schmidt, Patrick Richter, Sara Wright, Eike Zimmermann, Yan Ley, Julia van der Meer, Thomas Hartsch, Christian Bernloehr, Kerstin Otte, Harald Bradl, Martin Gamer, Patrick Schulz

**Affiliations:** ^1^ Cell Line Development, Bioprocess Development Biologicals Boehringer Ingelheim GmbH & Co. KG Biberach Germany; ^2^ Early Stage Bioprocess Development, Bioprocess Development Biologicals Boehringer Ingelheim GmbH & Co. KG Biberach Germany; ^3^ Institute of Applied Biotechnology University of Applied Sciences Biberach Germany; ^4^ Process Science Boehringer Ingelheim Fremont Inc. Fremont California USA; ^5^ Analytical Science Boehringer Ingelheim Fremont Inc. Fremont California USA; ^6^ Genedata AG Basel Switzerland; ^7^ Protein Science, Bioprocess & Analytical Development Boehringer Ingelheim GmbH & Co. KG Biberach Germany

**Keywords:** Chinese hamster ovary (CHO) cells, microRNA, monoclonal antibody, N‐glycosylation, sialylation

## Abstract

Chinese hamster ovary (CHO) cells are known not to express appreciable levels of the sialic acid residue N‐glycolylneuraminic acid (NGNA) on monoclonal antibodies. However, we actually have identified a recombinant CHO cell line expressing an IgG with unusually high levels of NGNA sialylation (>30%). Comprehensive multi‐OMICs based experimental analyses unraveled the root cause of this atypical sialylation: (1) expression of the cytidine monophosphate‐N‐acetylneuraminic acid hydroxylase (*CMAH*) gene was spontaneously switched on, (2) *CMAH* mRNA showed an anti‐correlated expression to the newly discovered *Cricetulus griseus* (cgr) specific microRNA *cgr*‐miR‐111 and exhibits two putative miR‐111 binding sites, (3) miR‐111 expression depends on the transcription of its host gene SDK1, and (4) a single point mutation within the promoter region of the sidekick cell adhesion molecule 1 (*SDK1*) gene generated a binding site for the transcriptional repressor histone H4 transcription factor *HINF‐P*. The resulting transcriptional repression of *SDK1* led to a downregulation of its co‐expressed miR‐111 and hence to a spontaneous upregulation of *CMAH* expression finally increasing NGNA protein sialylation.

## Graphical abstract

Fischer and co‐workers identified a mAb producing CHO cell line exhibiting unusually high levels of potentially immunogenic NGNA sialylation. A comprehensive multi‐OMICS based root cause analysis revealed that the *CMAH* gene was upregulated as a result of a point mutation identified in the promoter region of the *SDK1* gene which led to the silencing of its intronic miR‐111. MiR‐111 has been discovered as a new regulatory RNA in CHO cells responsible for *CMAH* gene and thus sialylation regulation.

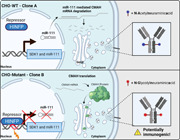

**Abbreviations**
CMAH: cytidine monophosphate (CMP)‐N‐acetylneuraminic acid hydroxylase
CNVs: Copy Number Variations
DMB: 1,2‐Diamino‐4,5‐methylenedioxybenzene dihydrochloride
FNIII: fibronectin type III
Gfi‐1: Growth Factor Independent‐1
GLM: Generalized Linear Model
HINF‐P: sidekick cell adhesion molecule 1; histone H4 transcription factor
NANA: N‐acetylneuraminic acid
NGNA: N‐glycolylneuraminic acid
sgRNAs: Single guide RNAs
ST6GAL: α‐2,6‐sialyltransferases

## 1 INTRODUCTION

N‐glycosylation represents one of the most important product quality attributes of therapeutic glycoproteins such as monoclonal antibodies (mAbs) (Walsh & Jefferis, 2006; Zhou & Qiu, 2018). The composition of the N‐linked glycan structure influences the efficacy of the mAb since effector functions such as antibody‐dependent cellular cytotoxicity and the complement‐dependent cytotoxicity are dependent on the glycosylation pattern (Mimura et al., 2016). Furthermore, the type of sugar moiety is also critical for the safety and/or serum half‐life of a glycoprotein in the blood (Zhou & Qiu, 2018). Unlike other glycoproteins, the N‐linked glycans of mAbs are predominantly bi‐antennary complex structures with terminal sialylation as the most advanced glycosylation form (Wang et al., 2018). In mammalian cells, two different types of terminal sialic acids are commonly found, the N‐acetylneuraminic acid (NANA) and its hydroxylated form N‐glycolylneuraminic acid (NGNA) (Hossler et al., 2009). In humans, only the NANA sialylation is present on glycoproteins since humans exhibit a homozygous loss‐of‐function mutation in the cytidine monophosphate (CMP)‐N‐acetylneuraminic acid hydroxylase (*CMAH*) gene catalyzing the conversion from CMP‐NANA into CMP‐NGNA (Chou et al., 1998; Okerblom et al., 2017; Varki, 2007). Hence, glycoproteins exhibiting NGNA sialylation are suspected to have high immunogenic potential and thus need to be avoided on biopharmaceutical products (Ghaderi et al., 2010).

Chinese hamster ovary (CHO) cells are the predominant expression host for recombinant human therapeutic glycoproteins (Durocher & Butler, 2009; Omasa et al., 2010). Besides many other important advantages, CHO cells are capable of producing recombinant glycoproteins with human‐like N‐glycosylation pattern. Notably, unlike other nonhuman mammalian expression hosts such as mouse myeloma NS0 or SP2/0 cell lines, this similarity also includes a very low abundance of terminal NGNA sialylation (<2%) (Hokke et al., 1990; F. Li et al., 2010), making CHO cells an attractive and safe manufacturing host for therapeutic glycoproteins. The reason for the low abundant NGNA levels on glycoproteins produced by CHO cells is that the CMAH protein is not expressed in this host, although an intact *CMAH* gene has been identified on the CHO genome (Xu et al., 2011). It is currently still unknown why the *CMAH* gene is silent in CHO cells, but we have recently generated a recombinant CHO DG44 cell line expressing a mAb that exhibited unusually high levels of NGNA sialylation both on the secreted mAb (>30%) as well as on cell surface glycoproteins. In this study, we provide several lines of evidence that the loss of expression of a newly identified small noncoding RNA (*cgr*‐miR‐111) was finally responsible for an upregulation of the *CMAH* gene leading to increased NGNA sialylation levels. Furthermore, we demonstrated that loss of expression of miR‐111 was induced by a single point mutation in the promoter region of the miR‐111 host gene sidekick cell adhesion molecule 1 (*SDK1)*, which gave rise to a binding site of an active transcriptional repressor histone H4 transcription factor (*HINF‐P*). The resulting silencing of *SDK1* and miR‐111 led to a deregulation of *CMAH* expression finally inducing the increased NGNA sialylation of the recombinant mAb.

## 2 MATERIALS AND METHODS

### 2.1 Cell culture

Two different recombinant CHO DG44 production clones (Clones A and B) expressing an identical monoclonal antibody (IgG4 isotype) were routinely cultivated in shake flasks (Corning) unless otherwise stated. Both cell lines were generated in the scope of a cell line development program and thus derived from the same transfection pool of suspension adapted Boehringer Ingelheim (BI) proprietary DHFR deficient (DHFR^–/–^) CHO DG44 host cells (for more details on the cell line development process please refer to Section 2.3). Cells were cultivated at 37°C, 5% CO_2_ and with agitation at 125 rpm (50 mm orbit) in an orbital shaker incubator (Infors). BI proprietary serum‐free, chemically defined and animal component free cell culture media were used for cultivation. Seeding cell density of stock cultures was set to 0.3 × 10^6^ viable cells per milliliter and cells were passaged every 3–4 days. Cell concentration and viability during routine stock culture cultivation was assessed using a Cedex HiRes Analyzer™ (Roche Diagnostics) by means of trypan blue exclusion.

### 2.2 Transfection

Transfection of small RNAs such as miRNA mimics or siRNAs (Qiagen) was performed as previously described using ScreenFect® A plus (InCella) (Fischer et al., 2013). Briefly, ScreenFect® A plus was first diluted in ScreenFect® Dilution Buffer (InCella) before the respective amount of miRNA or siRNA (50 nM final RNA concentration) was added followed by a complexation period of 20 min at room temperature. Exponentially growing CHO cells were pelleted, resuspended in fresh culture medium and added to each well containing transfection complexes at a cell concentration of 0.5 × 10^6^ viable cells per milliliter. Duetz® 6‐deepwell culture plates (Enzyscreen) were cultivated in an orbital shaker incubator (Infors) at 37°C, 5% CO_2_, 90% relative humidity and with agitation at 220 rpm (50 mm orbit).

Transfection of plasmid DNA was performed using the Cell Line Nucleofector® Kit V (Lonza) on a Nucleofector® (Lonza) according to the instructions provided by the manufacturer. Briefly, 5.0 × 10^6^ cells were pelleted and resuspended in transfection solution. After addition of 5 µg of endotoxin‐free plasmid DNA cells were transfected and seeded in pre‐warmed culture medium. Cell culture medium was exchanged for selection medium 24 h post transfection followed by a selection period of 2 weeks. Transfected cells were cultivated at 37°C and 5% CO_2_ in humidified atmosphere until single cell cloning. Each transfection was performed in biological triplicates (*n* = 3).

### 2.3 Cell line development (CLD)

Stable mAb producing CHO cell lines were generated by transfection of BI proprietary DHFR deficient (DHFR^−/^^−^) CHO‐DG44 host cells with 5 µg of two expression plasmids encoding the heavy and light chain genes of an IgG4 monoclonal antibody, respectively. Transfection was performed as described above. Stable recombinant cell pools were subjected to a stepwise gene amplification procedure by supplementation of the culture medium with increasing concentrations of methotrexate (MTX). Stable gene‐amplified pools were used for single cell cloning by fluorescent activated cell sorting (FACS) on a FACSAria™ III instrument (BD Biosciences). Approximately 6000 cell clones were deposited in multiple 384‐well plates (Corning) and cultivated for additional 14 days in BI proprietary single cell cloning medium. Automated fluorescence microscopy was conducted on Days 0, 7, and 14 post cloning using a Cellavista™ high‐end RS cell imager (Synentec bio Services) to assure generation of monoclonal cell lines as well as to monitor cell growth post cloning. After determination of accumulated mAb titers in the culture supernatant the clones exhibiting the highest mAb productivity were expanded to shake flasks and cryopreserved in safety cell banks.

### 2.4 Fed‐batch cultivation

Analysis of bioprocess performance of the two investigated stable CHO production cell lines was performed in controlled fed‐batch cultivations using 2L glass bioreactors. Briefly, cells were seeded at 0.3 × 10^6^ viable cells per milliliter in a BI proprietary production cell culture medium. Feeding was started at Day 3 following inoculation using a proprietary feed medium and included 30 ml of feed medium per liter of cultivation volume per day. Glucose levels were kept above 3 g/L throughout the entire cultivation period. Process parameters (cell concentration, viability, pH, pO_2_, glucose, and lactate concentration) as well as mAb concentration were determined daily. Cell concentration and viability was analyzed using a Cedex HiRes Analyzer™ (Roche Diagnostics). Glucose and lactate concentration was determined on a Biosen C‐Line System (EKF Diagnostic), while pH and pO_2_ were analyzed using a RAPIDLab®248 system (Siemens Healthcare). Antibody concentration was determined as described below (2.6) using a FortéBio Octet® HTX system (Pall Life Science).

### 2.5 Quantitative reverse‐transcription real‐time PCR (qRT‐PCR)

Total RNA was extracted from 5.0 × 10^6^ cells using the miRNeasy Mini Kit (Qiagen) according to the manufacturer's protocol. RNA concentration was measured on a NanoDrop spectrophotometer (Thermo Fisher Scientific) and 10 ng of RNA was reverse‐transcribed into cDNA using the TaqMan microRNA Reverse Transcription Kit (Thermo Fisher Scientific, #4366596) according to the manufacturer's instructions. Quantification of mature miRNAs was performed using the TaqMan™ Advanced miRNA Assay (Thermo Fisher Scientific, #A25576) on a Cfx96 instrument (Biorad). U6 snoRNA was used as reference small RNA. Calculation of ΔCq values was performed with the single threshold method (Biorad CFX manager software 2.1).

### 2.6 Monoclonal antibody quantification

Product concentration of mAbs was determined via bio layer interferometry using Protein A coupled biosensors on an FortéBio Octet® HTX system (Pall Life Science). Cell culture supernatant was diluted in cultivation media to a total volume of 200 µl. The Protein A coupled biosensors were incubated in cultivation media for 15 min for equilibration. Recuperation of biosensors between each measurement was achieved in a regeneration solution containing 10 mM glycine at a pH of 1.5. A standard curve using the respective purified monoclonal antibody was used to calculate product concentrations in cell culture supernatant.

### 2.7 Antibody sialylation analysis

Sialic acids were released from monoclonal antibody samples by acid hydrolysis and labeled with 1,2‐Diamino‐4,5‐methylenedioxybenzene dihydrochloride (DMB) using the Prozyme Glyko® Signal™ DMB Sialic Acid Labeling Kit. Standard solutions were prepared using NANA and NGNA purchased from Sigma Aldrich. DMB‐labeled sialic acids were separated by HPLC using a Prozyme GlycoSep™ R HPLC column and a Waters® 2795 HPLC system using an isocratic elution with 7% acetonitrile and 5% methanol. Separated DMB‐labeled sialic acids were detected by fluorescence with excitation at 373 nm and emission at 448 nm. Quantification of NGNA and NANA sialic acid was performed by interpolation of the fluorescence signal to a calibration curve for each individual sialic acid, spanning an on‐column load of approximately 0.1–3 ng.

### 2.8 Next‐Generation sequencing

RNA was extracted with the QIAsymphony® system using a QIAsymphony® RNA Kit (Qiagen). Library generation with 250 ng input RNA was performed using the TruSeq Stranded mRNA Library Prep Kit (Illumina, Inc.) according to the manufacturer's recommendations. Qualification of RNA and sequencing libraries was performed using an Advanced Analytical Fragment Analyzer™ (Advanced Analytical Technologies, Inc, Heidelberg, Germany) with High Sensitivity RNA Analysis DNF‐472 (15 nt) and Standard Sensitivity NGS Fragment Analysis DNF‐473 Kits. Quantification of sequencing libraries was carried out on a Tecan Infinite® 200 Pro Reader (Tecan Group) using the Quant‐iT™ PicoGreen® dsDNA Kit (Invitrogen). Libraries were pooled and sequenced on a HiSeq. 3000 instrument (Illumina, Inc.) in single‐end mode and 85 cycles.

### 2.9 DNASeq raw data processing

Genomic DNA of the two different CHO production cell lines (Clones A and B) at two different cultivation time points (D1 and D9) was sequenced with Sanger/Illumina 1.9 TruSeq PCR‐free gDNA (125 bp paired‐end reads and 350 bp insert size). Quality was assessed using FastQC (v0.11.5). Samples were quality trimmed with trimmomatic (v0.32) (Bolger et al., 2014). Quality‐trimmed libraries were normalized to the size of the smallest library to avoid library‐size dependent discrepancies in downstream processing. Reads were mapped against the reference genome (Brinkrolf et al., 2013) using bowtie2 to produce clone and day specific assemblies (Clone_A_D1, Clone_A_D9, Clone_B_D1, and Clone_B_D9) (v. 2.2.5) (Langmead & Salzberg, 2012). To capture cell line specific genomic DNA, paired‐end reads not mapping to the reference genome were de novo assembled using spades (v3.7.1) (Nurk et al., 2017). Assembly statistics was assessed with quast (v4.2) (Gurevich et al., 2013). Scaffolds smaller than 1000 bps were removed from the assemblies. Mutations were identified and quality filtered using Genedata Selector® Processor Module. Based on the mutation lists and the reference genome, fully annotated proprietary genomes including the mutation information were created using Genedata Selector® Explorer backend tool straincreator. CNVs were identified and quality filtered using Genedata Selector® Processor Module.

### 2.10 Generation of a cell line specific reference

The Chinese hamster genome (Brinkrolf et al., 2013) was used as the basic reference genome and integrated to Genedata Selector®. For the generation of a cell line specific reference, the Chinese hamster genome was enhanced with genomic DNA specific for the cell lines of this study to produce an intermediate reference C_griseus_adapted: (1) The two expression plasmids containing heavy and light chain genes, respectively, of the monoclonal antibody used in this study; (2) De novo assembled scaffolds of raw reads from this study not mapping to the Chinese hamster genome nor to the expression plasmids. The DNASeq raw reads from this study were then mapped to the intermediate reference C_griseus_adapted. Based on the mutation list from Clone_A_day_1 (sample from Clone A on Day 1 of the fed‐batch fermentation run) and the C_griseus_adapted genome, a fully annotated Clone_A_day_1 genome was generated which served as final reference for the RNASeq and miRNASeq data analyses. Whole‐genome promoter regions and transcription factor binding sites were in silico predicted based on position weight matrices from JASPAR (Fornes et al., 2020) using a Genedata Selector® algorithm (promoterSearch).

### 2.11 Verification of mutations and copy number variations (CNVs) in the *CMAH* locus

To verify that the *CMAH* gene locus shows no mutations or CNVs explaining the differences in expression between Clones A and B, the quality‐trimmed and library‐size‐normalized DNASeq raw data were also mapped to the relevant contig of the CHO Horizon assembly (Eagle Genomics Ltd [2017], Genbank assembly GCA_900186095).

### 2.12 RNASeq and miRNASeq data processing

For RNASeq analysis, samples from fed‐batch cultivation runs were sequenced with Sanger/Illumina TruSeq Stran mRNA (75 bp single‐end reads). Samples were quality trimmed with trimmomatic (v0.32) (Bolger et al., 2014). Reads were mapped against the Clone_A_day_1 reference genome using STAR (v2.52b) (Dobin et al., 2013). Mapped reads were quantified to raw read values per gene based on the refined gene model using Genedata Selector® Processor Module.

For miRNASeq analysis, samples from fed‐batch cultivation runs were sequenced with Sanger/Illumina TruSeq Small RNA (50 bp single‐end reads). Illumina Small RNA Adapters were removed with Cutadapt (v1.14) (Martin, 2011). Mature miRNAs were de‐novo predicted per sample using miRDeep* (v37.0) (An et al., 2013), without providing input annotation. For the mature miRNAs, miRNA target sites were predicted using TargetScan (v7.1) (Agarwal et al., 2015) with 3′UTRs deduced from the gene model of the Clone_A_day_1 reference.

### 2.13 Statistical analysis

To obtain fold factor and multiple testing corrected significance (BHQ) values between Clones A and B, we used DESeq. 2 (Love et al., 2014) with factor clone (normalization method: Regularized Log Transformation, Likelihood Ratio Test, omitting 20% most low‐abundant genes for RNASeq data and miRNASeq data, respectively). To test if the effects we identified for factor clone in the DESeq. 2 based analysis were time independent, we ran a Generalized Linear Model (GLM) for count data, assuming negative binomial data distribution (factors cell line and co‐variate time) using Genedata Selector® Analyst Module. PCA and Correlation analyses were performed on DESEq. 2 normalized (Regularized Log Transformation) data using Genedata Selector® Analyst Module.

### 2.14 Targeted genome editing of Clone B using Cas9

Cas9 was used to edit the genomic locus of the *CMAH* gene in the investigated CHO production cell line (Clone B) to confirm the catalytic activity of CMAH to be causative for the modified sialylation. Single guide RNAs (sgRNAs) were designed to introduce a frameshift mutation (InDel) using Genedata Selector® software, leading to a functionally inactive CMAH protein in Clone B. The plasmid backbone pX458 was used as Cas9 expression construct and source for one or two sgRNAs which were ordered as gBlocks™ (IDT Inc.), and cloned as described elsewhere (Ran et al., 2013). Twenty‐four hour post transfection, respective cell pools were subjected to single cell cloning by depositing GFP‐positive clones into 384‐well plates, as described above. Clonal cell lines were expanded and screened via PCR and Surveyor assay (IDT) and positive as well as control clones further investigated for altered surface protein sialylation via flow cytometry. In addition, Cas9 was used for targeted genome editing of a putative HINF‐P transcription factor binding site as well as for the deletion of the transcription factor HINF‐P in Clone B. Therefore, Cas9‐sgRNA ribonucleoprotein complexes (IDT) were transfected by electroporation into Clone B. The editing efficiency in the generated pool was assessed by PCR 3 days after transfection and the effect on surface glycoprotein NGNA sialylation was analyzed by flow cytometry 6 days post transfection. All utilized sgRNAs and PCR primers are summarized in Tables S1 and S2.

### 2.15 Flow cytometry mediated analysis of cell surface glycoprotein sialylation

To functionally study the effect of a genomic knockout of the *CMAH* gene in Clone B, CRISPR/Cas9 genome edited cell lines as well as control cells were examined for cell surface protein NGNA sialylation using the Anti‐Neu5Gc Antibody kit (Biolegend) according to the vendor protocol, and in combination with a donkey anti‐chicken IgY‐AlexaFluor647® conjugated secondary antibody (JacksonImmunoResearch). Cells were analyzed on a MACSQuant® Analyzer (Miltenyi Biotech).

## 3 RESULTS

### 3.1 Differential gene expression reveals CMAH to be causative for enhanced NGNA sialylation

**Figure 1:**
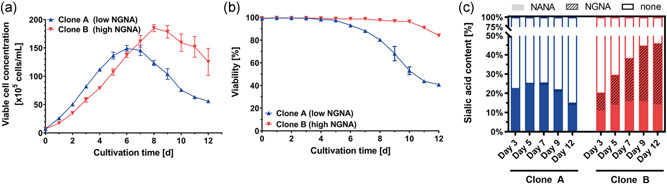
Monoclonal antibody production process of investigated Chinese hamster ovary (CHO) cell lines. (a) Viable cell concentration, (b) viability, and (c) secreted antibody sialylation (N‐acetylneuraminic acid (NANA) and N‐glycolylneuraminic acid (NGNA)) of the investigated recombinant CHO cell lines Clones A and B during a 2L fed‐batch cultivation. Cultivation data are presented as mean ± standard deviation of biological replicates. Harvested cell culture fluid was pooled prior to mAb purification and sialic acid quantification

Over the course of a routine CLD process to generate a recombinant mAb producing CHO cell line, one of the established stable cell lines (Clone B) was identified to express the desired IgG4 antibody with unusually high levels of terminal NGNA sialic acid. To systematically investigate this atypical glycosylation pattern, we initiated a comparative analysis of Clone B with one of its sister clones (Clone A), which was derived from the same CLD campaign but exhibited only trace levels of NGNA sialylation on the recombinantly expressed IgG4 antibody. Both clonal cell lines were cultivated in a controlled fed‐batch cultivation process using 2L bioreactors and demonstrated a similar growth behavior in the first half of the cultivation process, which changed from Day 6 onward (Figure 1a,b). Secreted antibodies were purified via Protein A chromatography followed by sialylation analysis of the purified mAbs (Figure 1c). On the day of the bioreactor harvest NANA sialylation levels seemed comparable between Clones A and B. However, mAbs produced by Clone B showed nearly 10‐fold increased NGNA sialylation at an absolute amount of ~35% of the secreted antibodies.

**Figure 2:**
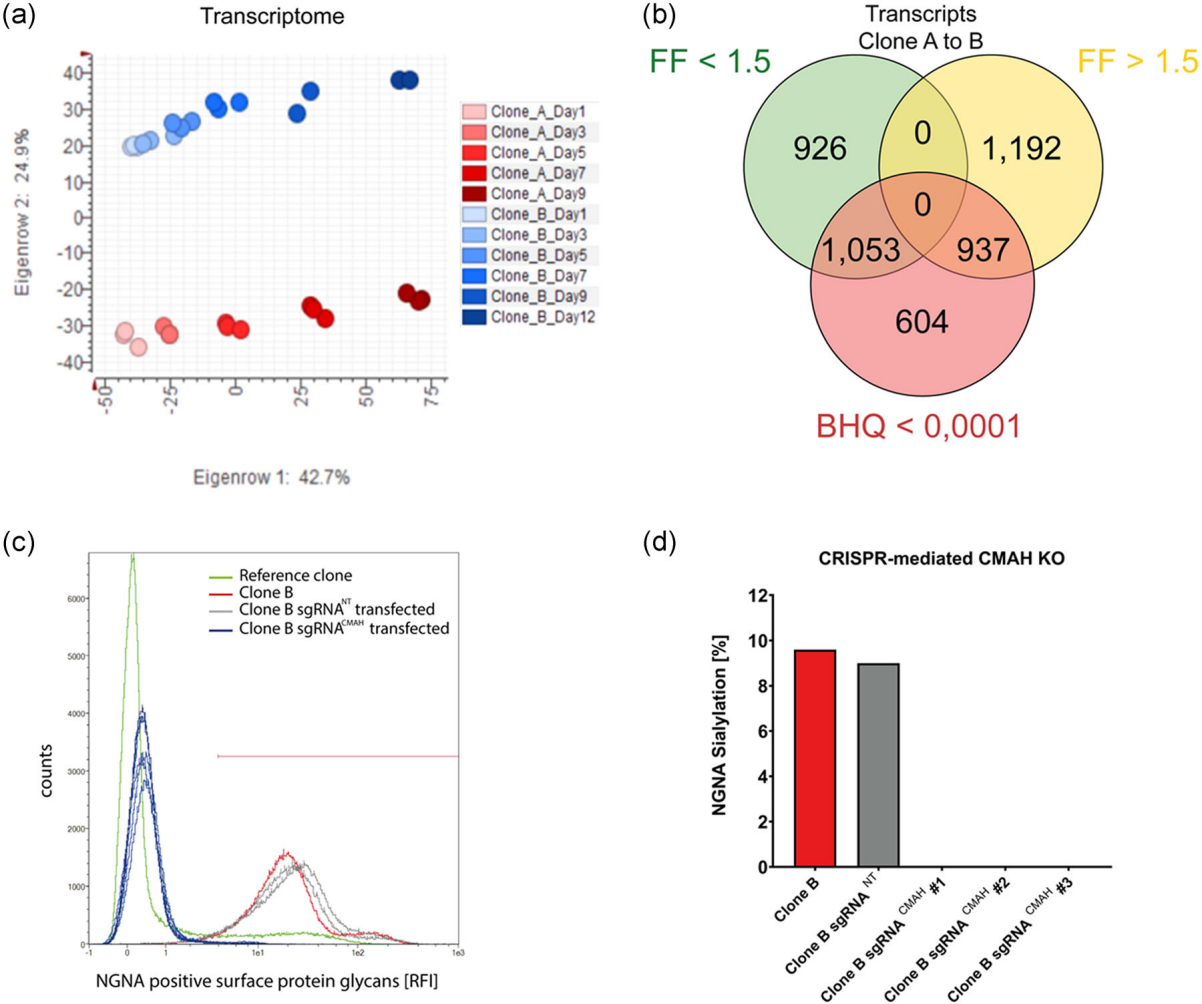
Differential gene expression identified cytidine monophosphate (CMP)‐N‐acetylneuraminic acid hydroxylase (CMAH) to be the molecular root cause of increased monoclonal antibody N‐glycolylneuraminic acid (NGNA) sialylation. (a) Principal component analysis (PCA) of transcriptomes (RNASeq) of Clones A and B. PCA based on Covariance Matrix of DESeq. 2 normalized data. (b) Differential analysis of factor Clone using DESeq. 2: genes with statistical significance for factor Clone of BHQ < 1E‐5 and FF > 1.5. (c) Flow cytometry analysis to determine cell surface protein NGNA sialylation of wildt‐ype Clone B (red line), three different negative CRISPR/Cas9 control (sgRNA^NT^) cell lines (gray lines), five different CMAH knockout clones (sgRNA^CMAH^) derived from Clone B (blue lines) and a reference CHO production cell line expressing a different monoclonal antibody (green line). An anti‐NGNA fluorescence staining kit was used for cell surface protein analysis. (d) NGNA sialylation of the secreted IgG4 monoclonal antibody expressed by either wildtype Clone B, negative CRISPR/Cas9 control cells (sgRNA^NT^) or three different CMAH knockout clones (sgRNA^CMAH^) derived from Clone B

To identify the root cause of the phenotypic difference between Clones A and B, genome, transcriptome and miRnome data were generated using Next‐Generation Sequencing (NGS). For these analyses, 31 samples were taken from the fed‐batch cultivations on Days 1, 3, 5, 7, 9, and 11 (Day 11 only for Clone B due to a low cell viability of <60% for Clone A) and used for preparation of RNASeq and miRNASeq libraries including biological replicates. Global variance in transcriptome data was visualized in a principal component analysis (PCA) (Figure 2a). Gene expression profiles were found to depend on the factors “Clone” and “Time.” Importantly, the factors seemed to be unrelated which was corroborated by a GLM for count data with factor “clone” and co‐factor “day” (data not shown). To identify differentially expressed genes, the time‐resolved gene‐level count tables were analyzed with DESeq. 2 for the factor clone (Figure 2b). One thousand nine hundred and ninety genes were differentially expressed between the two clones considering a fold change of >1.5 and a BHQ value of <0.0001 (1053 upregulated in Clone B and 937 genes upregulated in Clone A). These included *CMAH*, which is known to convert CMP‐NANA to the nonhuman and potentially immunogenic sialic acid precursor CMP‐NGNA in the cytosol (Ghaderi et al., 2010; Y. Li & Chen, 2012). Interestingly, *CMAH* showed a strong regulation between Clones A and B (Fold factor 8.9) and thus a relevant absolute expression of 12,441 raw read counts in Clone B.

CRISPR/Cas9 genome editing tools were used to investigate whether the CMAH protein was responsible for increased protein NGNA sialylation by modification of the genomic *CMAH* gene locus to produce a catalytically inactive variant. Generated clonal cell lines were subsequently investigated for global surface glycoprotein NGNA sialylation (Figure 2c). In contrast to the controls, Clone B derived cell lines with confirmed genomic modifications of *CMAH* displayed no surface glycoprotein NGNA sialylation anymore. In addition, compared to the controls the *CMAH* knockout clones had entirely lost the NGNA sialylation on the secreted IgG4 antibody (Figure 2d). This indicated that the catalytic activity of the CMAH protein was solely required for the upregulated NGNA protein sialylation and thus represented the causative effector protein for NGNA sialylation in Clone B. However, based on whole genome re‐sequencing data generated for both cell lines, we found neither copy number variations nor mutations for any of the two time points for Clone B relative to Clone A (data not shown). Therefore, we concluded that changes in *CMAH* levels between the two cell lines could not be explained by mutations in the coding region, promoter or 3′‐UTR of the *CMAH* gene itself or by a gene amplification event. We completed the analysis of the *CMAH* gene promoter region by bisulfite sequencing experiments that did not show differential DNA methylation either (data not shown).

### 3.2 Newly identified miR‐111 regulates the expression of CMAH

**Figure 3:**
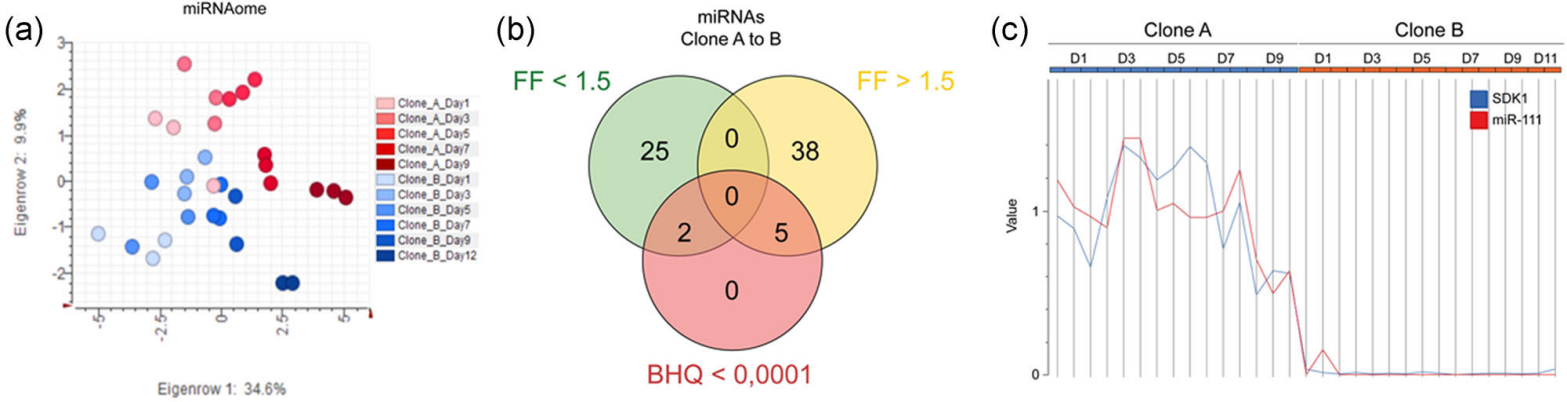
miRNA sequencing identified the Chinese hamster ovary specific miR‐111. (a) Principal component analysis of microRNAomes (miRNASeq analysis) of Clones A and B based on Covariance Matrix of DESeq. 2 normalized data. (b) Differential analysis of factor Clone using DESeq. 2: genes with statistical significance for factor Clone of BHQ < 1E‐5 and FF > 1.5. (c) Expression of miR‐111 (red) and its host gene *SDK1* (blue, shown are library size scaled raw data relative to the average expression of Clone A). *SDK1* is the gene with the highest correlation to miR‐111 (DESeq. 2 normalized value based *R*‐value of 0.96, *p*‐value < 1E‐16)

To investigate the regulation of *CMAH* gene expression, miRNASeq libraries were generated and mapped against Clone_A_D1 using miRDeep* (mapping efficiency of 2.3%, 1.15 million mapped read), resulting in 605 de novo predicted miRNAs quantified as raw read counts (Zit miRDeep*) (An et al., 2013). The variance in microRNAome data was visualized in a PCA (Figure 3a). Seven miRNAs were detected to be significantly differentially expressed considering a fold change of >1.5 and a BHQ value of <0.0001 (Figure 3b). Here, the miRNA with the strongest regulation was miR‐111 (fold factor 0.0297 Clone B vs. Clone A), which was further verified using qRT‐PCR analysis (Figure S1). Interestingly, analysis of the cell line‐specific genomes showed that miR‐111 is located in the first intron of the *SDK1* gene, which was also amongst the most significantly regulated genes found between Clones A and B (fold factor 0.0077 Clone B vs. Clone A). Corroborating the hypothesis that miR‐111 might be regulated via the *SDK1* gene promoter, the significantly differentially expressed genes were analyzed for correlation with miR‐111 expression. Strikingly, *SDK1* showed the strongest correlation (*R*‐value = 0.96, Figure 3c).

**Figure 4:**
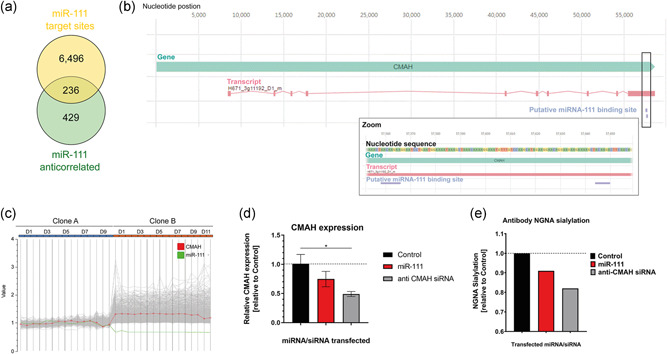
The newly identified miR‐111 regulates the expression of cytidine monophosphate (CMP)‐N‐acetylneuraminic acid hydroxylase (CMAH). (a) Six thousand seven hundred and thirty‐two genes show miR‐111 target site predictions (using TargetScan). Sequence based *cgr*‐miR‐111 target predictions are over‐represented in the 665 miR‐111 anti‐correlated genes (Fisher's exact test *p*‐value < 0.005). (b) *CMAH* gene locus in Clone A at Day 1 of the fed‐batch process with miR‐111 targets (predicted by TargetScan). Neither variants nor copy numbers explained the expression profile of *CMAH*. Zoom into miR‐111 target sites in *CMAH* 3′‐UTR region (c) 665 miR‐111 genes are anti‐correlated to miR‐111 (DESeq. 2 normalized value based *R*‐value < −0.75, *p*‐value < 1E‐5). Shown are DESeq. 2 normalized data relative to the average expression of Clone A. Note that the scale reflects logarithmic fold factor values. MiR‐111 is highlighted in green. *CMAH* is highlighted in red. (d) Relative *CMAH* mRNA expression 24 h after transient transfection with miR‐111 mimics (red), nontargeting control siRNA (black) or anti‐CMAH siRNA (gray). (e) N‐glycolylneuraminic acid sialylation of the secreted IgG4 monoclonal antibody at Day 6 after transient transfection with miR‐111 mimics (red), nontargeting control siRNA (black) or anti‐CMAH siRNA (gray), respectively

Assuming that miR‐111 played a key role in establishing the observed glycosylation phenotype of Clone B, we searched for potential miR‐111 targets in the CHO cell transcriptome. In total, 665 miR‐111 anti‐correlated transcripts with *R*‐values < −0.75 resulted from this analysis (Figure 4a). These transcripts were further investigated with respect to functional annotation (metabolic pathways, Pfam motifs and GO—biological process annotation). Twenty‐seven miR‐111 anti‐correlated genes showed functional annotation indicating a role in protein glycosylation (Table 1). Among the investigated metabolic pathways, “N‐glycan biosynthesis” was the pathway showing the highest over‐representation with a Fisher's Exact test *p*‐value < 0.01. Four genes were directly associated with the N‐glycan biosynthesis pathway. To analyze whether the miR‐111 anti‐correlated genes were potentially directly regulated by miR‐111, we conducted a genome‐wide in silico target site prediction and found miR‐111 target sites in the 3′‐UTRs of 6,732 genes. The 665 miR‐111 anti‐correlated genes showed a significant over‐representation (236 genes with target site predictions, Fisher's exact test *p*‐value < 0.005). Strikingly, *CMAH* was identified to exhibit two putative miR‐111 binding sites in its 3'UTR region (Figure 4b). One of them had the optimal predicted target sequence aACAAGAa. Moreover, *CMAH* and miR‐111 expression were significantly anti‐correlated (*R*‐value = −0.88, Figure 4c). To investigate whether miR‐111 directly regulates *CMAH* expression, we transiently transfected Clone B with miR‐111 mimics and found *CMAH* mRNA to be downregulated in comparison to the nontargeting control (Figure 4d). In line with the decreased *CMAH* mRNA expression also the NGNA sialylation of the secreted IgG4 antibody was reduced following transient transfection of miR‐111 mimics (Figure 4e). Thus, we concluded that *CMAH* is most likely negatively regulated by miR‐111 in CHO cells.

**Table 1:** Twenty‐seven miR‐111 anti‐correlated genes are found to be associated with protein glycosylation (via Metabolic pathway, GO—biological process and/or Pfam motif annotation)

Gene ID	Putative miR‐111 target	GO—biological process description	Correlation with miR‐111
*B3GALT5*	No	Protein glycosylation	−0.9502142
*BMPER*	No	Blood vessel endothelial cell proliferation involved in sprouting angiogenesis: positive regulation of ERK1 and ERK2 cascade	−0.8280621
*CLSTN2*	No	‐	−0.9454502
*CMAH*	**Yes**	**Protein glycosylation**	**−0.8793129**
*DAPK1*	Yes	Signal transduction: protein phosphorylation: positive regulation of apoptotic process	−0.9143201
*ENGASE*	No	‐	−0.7782611
*FUT2*	No	Carbohydrate metabolic process	−0.8837712
*ganab*	No	Carbohydrate metabolic process: metabolic process	−0.8397844
*GLYCAM1*	Yes	‐	−0.7507119
*H671_1g3041*	No	‐	−0.875597
*H671_21100*	No	‐	−0.8112577
*H671_3g9619*	Yes	Metabolic process	−0.9275447
*H671_4g11585*	Yes	Protein glycosylation	−0.8601853
*H671_6g15320*	Yes	‐	−0.922971
*IRAK1*	No	Protein phosphorylation: positive regulation of transcription from RNA polymerase II promoter	−0.7970828
*MMP9*	No	Positive regulation of keratinocyte migration: proteolysis: metabolic process	−0.8193061
*MYT1L*	No	Response to oxidative stress: oxidation‐reduction process: regulation of transcription, DNA‐dependent: multicellular organismal development	−0.8830791
*PIGC*	No	GPI anchor biosynthetic process	−0.8084261
*PLOD2*	No	‐	−0.7697681
*RASAL3*	No	Regulation of small GTPase mediated signal transduction: peptidoglycan catabolic process: signal transduction	−0.8330823
*SECTM1*	No	Positive regulation of I‐kappaB kinase/NF‐kappaB cascade	−0.8073307
*SLC22A14*	No	Transmembrane transport	−0.9027236
*SLC2A4*	No	Transmembrane transport: transport	−0.7896906
*ST3GAL4*	**Yes**	**Protein glycosylation**	**−0.7847602**
*ST6GAL1*	**Yes**	**Protein glycosylation**	**−0.7736951**
*SYT8*	No	‐	−0.9338071
*TMTC2*	Yes	‐	−0.8833522

*Note*: Genes involved in protein sialylation are highlighted in bold. Putative miR‐111 target: gene exhibits putative binding site in 3′UTR as identified using Target Scan.

### 3.3 Investigation of the miR‐111/SDK1 sequence and DNA locus discovers a critical sequence variation

**Figure 5:**
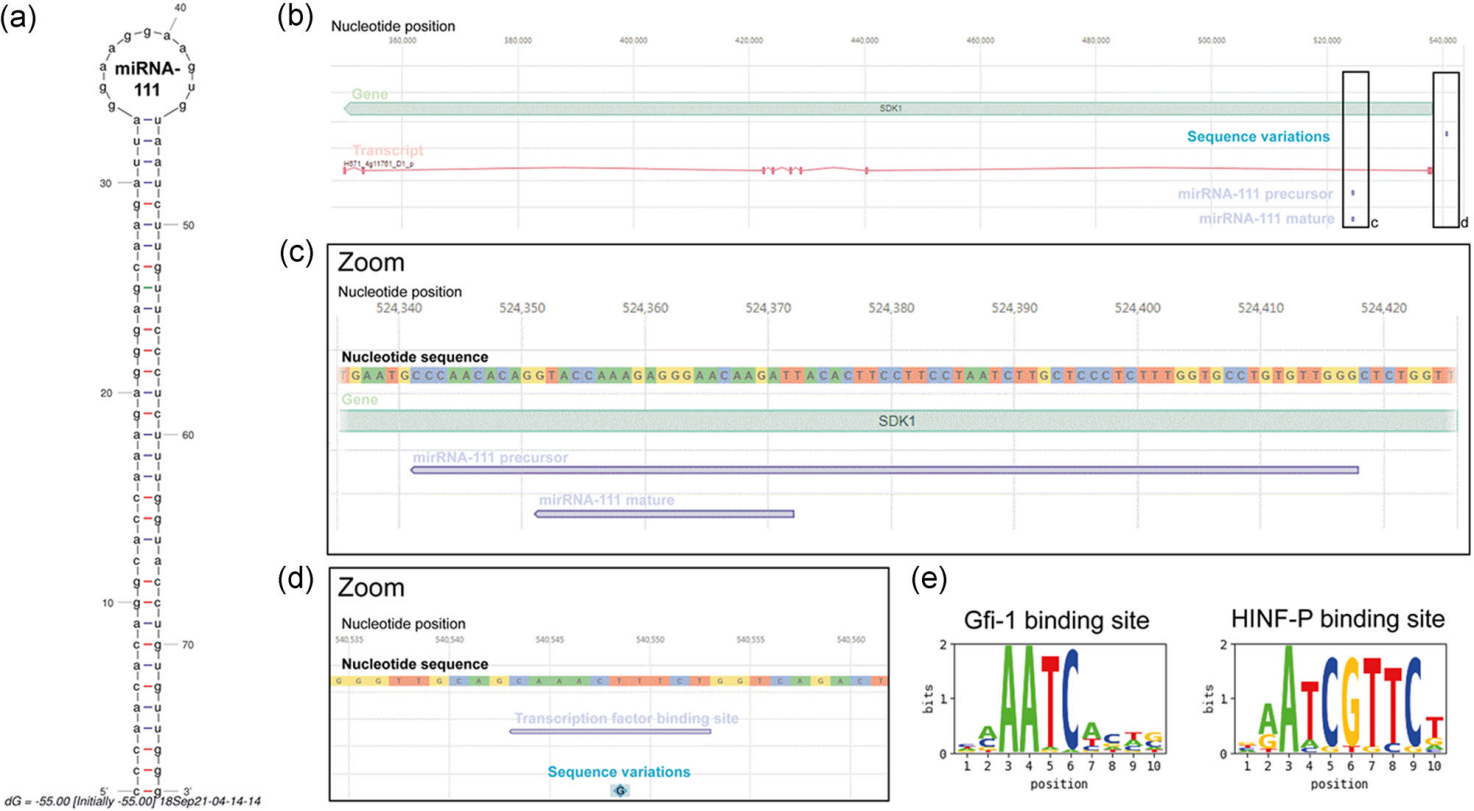
A sequence variation in the promotor region resulted in SDK1/miR‐111 silencing. (a) Secondary structure of the newly discovered miR‐111 precursor microRNA. (b) Schematic overview on the genomic locus of the *SDK1* gene. Shown are the location of the miR‐111 precursor and mature miRNA as well as the single nucleotide variant detected in the *SDK1* gene promoter region as compared to the genomic DNA of Clone A. MiR‐111 is located in the first intron of the *SDK1* gene. (c) Zoomed view into the DNA sequence of miR‐111 precursor and mature miRNA. (d) Zoomed view into the DNA sequence at the position where the single nucleotide variance was detected in Clone B in comparison to Clone A. The shown single point mutation occurred in Clone B at contig position 540549 (G instead of T). A putative HINF‐P binding site was predicted (2395–2404 bases upstream of the SDK1 transcription start site, ident score = 0.843, *p*‐value < 0.0001). In contrast, a Gfi‐1 binding site was predicted in this region for Clone A. Please note that the *SDK1* gene and miR‐111 binding sites are positioned on the DNA minus strand. (e) Gfi‐1 and HINF‐P transcription factor binding sites as predicted using position weight matrices from JASPAR via a Genedata Selector® algorithm (promoterSearch)

The nearly palindromic miR‐111 precursor sequence predicted by miRDeep* exhibits a very stable 2‐D structure (−55 kcal/mol, Figure 5a). The miRDeep* predicted mature miR‐111 sequence (aTCTTGTtccctctttggtac) has a TargetScan predicted optimal target sequence of aACAAGAa. We verified that the exclusive presence of miR‐111 in the CHO cell/Chinese hamster (*Cricetulus griseus*) genome was not previously reported by blasting both the precursor as well as the mature miR‐111 sequence against the mirBase (using blastn‐short, data not shown). After verification of miR‐111 as a true Chinese hamster (*cgr*)‐specific miRNA, we sought to pinpoint the reason for differential SDK1 regulation leading to the expression of miR‐111 and finally the changes in mAb sialylation. For this purpose, a detailed analysis of the *SDK1* gene locus was conducted (Figure 5b). MiR‐111 is located in the first intron of *SDK1*. Both *SDK1* and miR‐111 are positioned on the DNA minus strand. No mutations were identified within the first *SDK1* intron (Figure 5c). Postulating that *SDK1* is the host gene of miR‐111 and thus miR‐111 expression is controlled by the *SDK1* gene promoter, we investigated the *SDK1* gene promoter region for sequence variants between Clones A and B and indeed found a single point mutation (Figure 5d). Consequently, we performed a prediction of transcription factor binding sites in the *SDK1* promoter region (−5000 bp to +500 bp relative to the transcription start site) of both clones. A binding site of the zinc finger repressor protein growth factor independent‐1 (*Gfi‐1*) was predicted in the promoter of Clone A (2394–2403 bases upstream of the *SDK1* transcription start site, ident score = 0.709, *p*‐value < 0.0001). Due to a point mutation within exactly this binding site in Clone B (540549ptT > G), the Gfi‐1 binding site was predicted to be changed into a binding site of another repressor—HINF‐P—(2395–2404 bases upstream of the *SDK1* transcription start site, ident score = 0.843, *p*‐value < 0.0001) (Figure 5e). Interestingly, looking at the transcriptomic data of both clones *HINF‐P* shows a much higher overall mRNA expression than *Gfi‐1* (27,988 vs. 324 overall raw read counts, respectively) indicating that Gfi‐1 might not be expressed at all in CHO cells. Finally, CRISPR/Cas9 mediated genomic deletion of either the newly generated putative HINF‐P TF binding site or the *HINF‐P* transcription factor gene both led to a significant decrease in NGNA sialylation of surface glycoproteins in Clone B (Figure S2). Thus, the identified single point mutation (540549ptT > G) in the *SDK1* gene promoter very likely represents the root cause of changes in miR‐111 expression as well as its downstream gene targets between Clones A and B leading to the altered protein sialylation.

## 4 DISCUSSION

The absence of NGNA sialylation is of importance to biotherapeutics since it has been reported that circulating anti‐NGNA antibodies are present in human blood and thus the occurrence of NGNA sialylation on biologics may likely induce immunogenic reactions (Ghaderi et al., 2010; Tangvoranuntakul et al., 2003). Interestingly, a functionally intact *CMAH* gene was found in the CHO cell genome but no expression could be detected, which is consistent with the common understanding that CHO cells are producing mAbs with human‐like glycosylation (Xu et al., 2011). So far, the reason for the CMAH negative phenotype of CHO production cell lines remained unidentified. However, we were able to provide several lines of evidence that the newly discovered and CHO‐specific (*cgr*‐)miRNA‐111, which was identified to have two putative binding sites in the 3′UTR of the *CMAH* mRNA, might represent a strong permanent negative regulator of the *CMAH* gene and the first miRNA demonstrated to regulating parts of the N‐glycosylation pathway in CHO cells. As it turned out, miR‐111 showed decreased expression in an individual CHO production clone (Clone B). The reason for the altered *CMAH* expression could be traced back to an abrupt silencing of both miR‐111 and its host gene *SDK1* and thus an increased expression of the putative miR‐111 target gene *CMAH*.

SDK1 and its homolog SDK2 are among the largest within the group of immunoglobulin superfamily molecules, which facilitate cellular functions as surface receptors, co‐receptors, co‐effectors, or adhesion molecules (Yamagata, 2020). They share a highly conserved domain organization with a signal sequence, 6 immunoglobulin (Ig) domains, 13 fibronectin type III (FNIII) domains, a transmembrane domain, and a cytoplasmic part (Yamagata, 2020). In particular, the four N‐terminal Ig domains mediate specific cell–cell interactions between SDK positive cells facilitating, for example, synaptic layer specificity of ganglion cells during retina development (Goodman et al., 2016; Honig & Shapiro, 2020; Sanes & Yamagata, 1999, 2009; Yamagata, 2020). However, some FNIII domain containing proteins show lectin‐like properties with certain carbohydrate binding specificity (Kleene et al., 2001; Yamagata et al., 2002). The characteristic motif associated with a NANA(2,3)‐Gal carbohydrate binding capacity (Kleene et al., 2001) is also present in the Chinese hamster SDK1 protein sequence (data not shown). Interestingly, we identified the N‐glycosylation modifying miR‐111, favoring exclusive NANA sialylation, to be located within the first intron of the *SDK1* gene and being dependent on its transcriptional activity. Thinking of the specific interaction between SDK1 positive cells, the modulation of protein sialylation within these cells to principally NANA might further strengthen the interaction due to their recognition by the FNIII domains. However, the spontaneous silencing of miR‐111 synthesis in the investigated recombinant CHO cell line Clone B was likely initiated by a single nucleotide exchange in the upstream promoter region of its host gene *SDK1* changing a putative Gfi‐1 transcription factor binding site into a HINF‐P binding site.

Gfi‐1 is a transcriptional zinc finger protein belonging to the Snail/Gfi‐1 domain family and represses the expression of genes implicated in cell survival, proliferation, cell fate specification and differentiation (Chiang & Ayyanathan, 2013; Jafar‐Nejad & Bellen, 2004; van der Meer et al., 2010). Besides hematopoietic cell lineages and development, Gfi‐1 is also expressed in both the CNS as well as a variety of sensory organs (Jafar‐Nejad & Bellen, 2004). Especially during retina development, Gfi‐1 is required for terminal retinal ganglion cell differentiation and survival, which is in good agreement with a regulatory Gfi‐1 binding site upstream of the *SDK1* gene conferring synaptic layer specificity of some retinal ganglion cells by specific cell–cell interactions (Yamagata, 2020; Yang et al., 2003). Conclusively, Gfi‐1 has essential roles in hematopoietic cell and sensory organ development but its expression might not be required by CHO cells. However, a single point mutation in the respective transcription factor binding site upstream of the *SDK1* gene in the recombinant CHO cell line Clone B led to the conversion into a putative HINF‐P binding site. Despite of also being a transcriptional repressor, HINF‐P is a key regulator of multiple histone H4 genes, which are necessary during DNA replication and thus needs to be expressed in proliferating cells (Liu et al., 2011; Xie et al., 2009). In fact, *HINF‐P* showed much higher overall expression in CHO cells than *Gfi‐1* putatively leading to a downregulation of *SDK1* and its nested miR‐111 by the newly acquired recognition site in the recombinant CHO cell line Clone B.

Several genetic engineering strategies have been pursued in the past to modify N‐glycosylation pattern on recombinant proteins produced by CHO cells. In particular, sialic acid modulation and increasing galactosylation as an acceptor substrate for sialyltransferases were of major interest, due to its critical role in therapeutic glycoprotein half‐life and efficacy (Fischer et al., 2015; Tejwani et al., 2018; Wang et al., 2017). Even though the potentially immunogenic glycoepitope NGNA is usually not produced by CHO cells the corresponding gene has been knocked out as a precaution (Lin et al., 2013). Furthermore, CHO cells lack the expression of α‐2,6‐sialyltransferases (ST6GAL) and thus cannot produce glycoproteins with a similar terminal sialic acid content as compared to human cells which do express both α‐2,3‐ as well as α‐2,6‐ sialyltransferases (Bork et al., 2009; Butler, 2005; Jenkins et al., 1996; Xu et al., 2011). As a consequence, several recombinant CHO cells lines have been developed expressing different combinations of α‐2,6‐ and/or α‐2,3‐sialyltransferases with or without galactosyltransferases (Fischer et al., 2015; Tejwani et al., 2018; Wang et al., 2017). Interestingly, we identified that both *ST6GAL1* and *ST3GAL4* (being the most relevant ST3GAL for mAb sialylation) are also putative targets of miR‐111 and showed anti‐correlated expression to miR‐111 in Clone B. Therefore, a dual knockout in CHO cells leading to a deficiency in both miR‐111 and *CMAH* expression might result in significantly increased NANA sialylation abundance on glycoproteins. In addition, miR‐111 might still be useful as a surrogate marker for atypical NGNA sialylation in CHO cell lines in the future precluding the requirement for complex sialylation analytics. Of note, the superior growth performance observed for Clone B in the fed‐batch cultivation experiment is considered to be derived from clonal variances rather than induced by the loss of miR‐111/SDK1 expression since other well‐growing sister clones, which were established in the same CLD campaign, did not demonstrate increased NGNA sialylation levels.

As demonstrated in this study, the loss of expression of the newly discovered miR‐111 was finally responsible for an upregulation of the *CMAH* gene leading to increased NGNA sialylation of the secreted IgG4 antibody in a recombinant CHO production cell line, which could be traced back to a single point mutation in the promoter region of the *SDK1* gene. Hence, a new genetic regulatory circuit of protein sialylation in one of the industrially most relevant mammalian production cell systems could be revealed additionally explaining the reason for the so far unknown silencing mechanism of *CMAH* in CHO cells. Of note, miR‐111 has not been exclusively identified in CHO‐DG44 cells (from which Clone A and B were derived) but also in the CHO‐K1 genome (data not shown), indicating that this microRNA may play an important role in most industrially relevant CHO production cell lines used for biopharmaceutical production.

## CONFLICT OF INTERESTS

S. Fischer, S. Mathias, A. Stadermann, V. Schmieder, N. Schmidt, N. Zeh, P. Richter, C. Bernloehr, H. Bradl, M. Gamer, and P. Schulz are employees of Boehringer Ingelheim Pharma which develops and sells pharmaceuticals. The authors declare that there are no conflict of interests.

## Supporting information

Supporting information.

Supporting information.

Supporting information.

Supporting information.

## Data Availability

Data available on request from the authors.
