# Supplementary figures and images for: Loss of a newly discovered microRNA in Chinese hamster ovary cells leads to upregulation of N‐glycolylneuraminic acid sialylation on monoclonal antibodies
Source: Biotechnol Bioeng. 2022 Jan 14;119(3):832–44. doi: 10.1002/bit.28015 (PMC9306616; doi:10.1002/bit.28015)

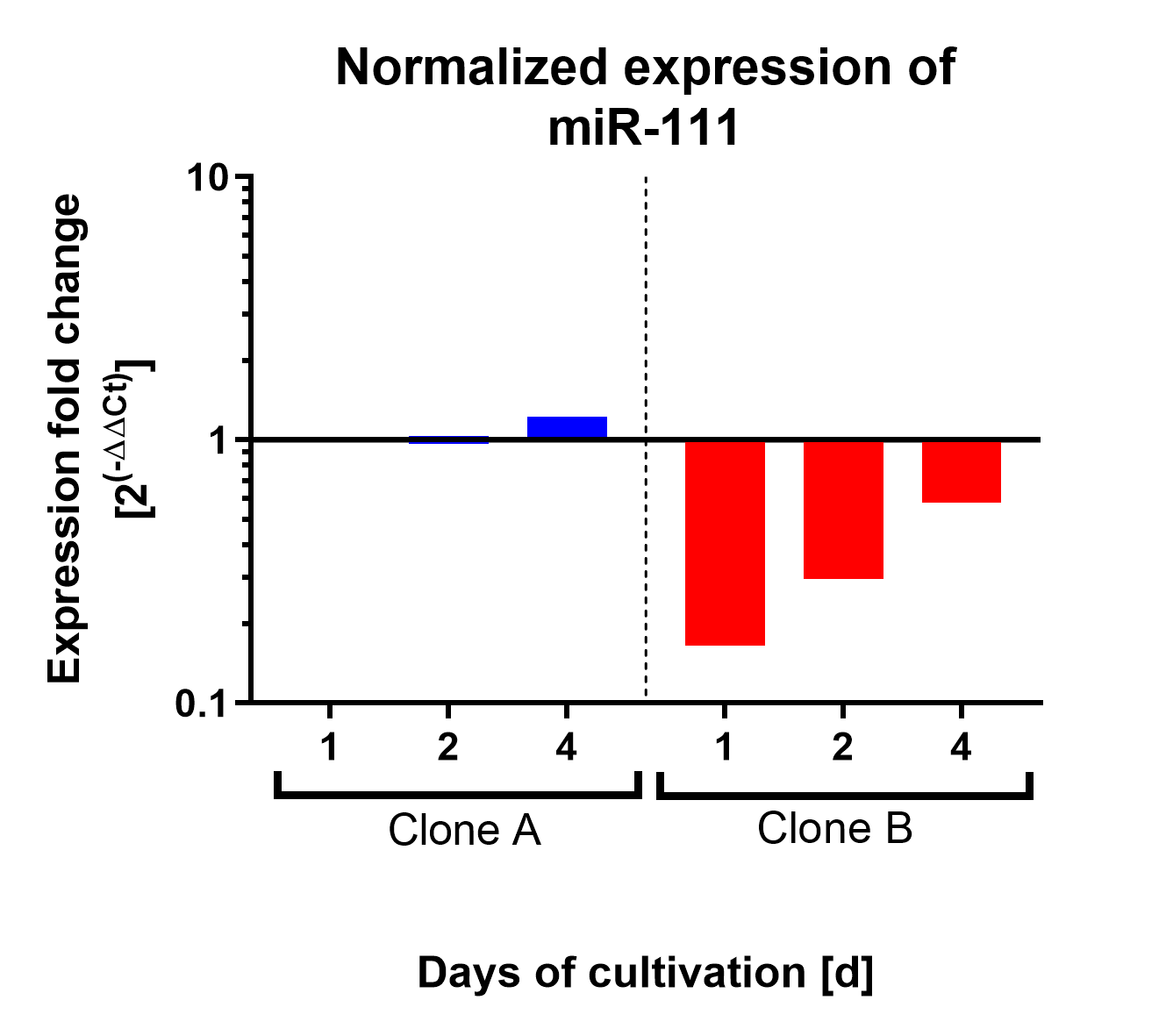

Supplement: Supplementary file 1 — Supporting information. [file BIT-119-832-s002.png]

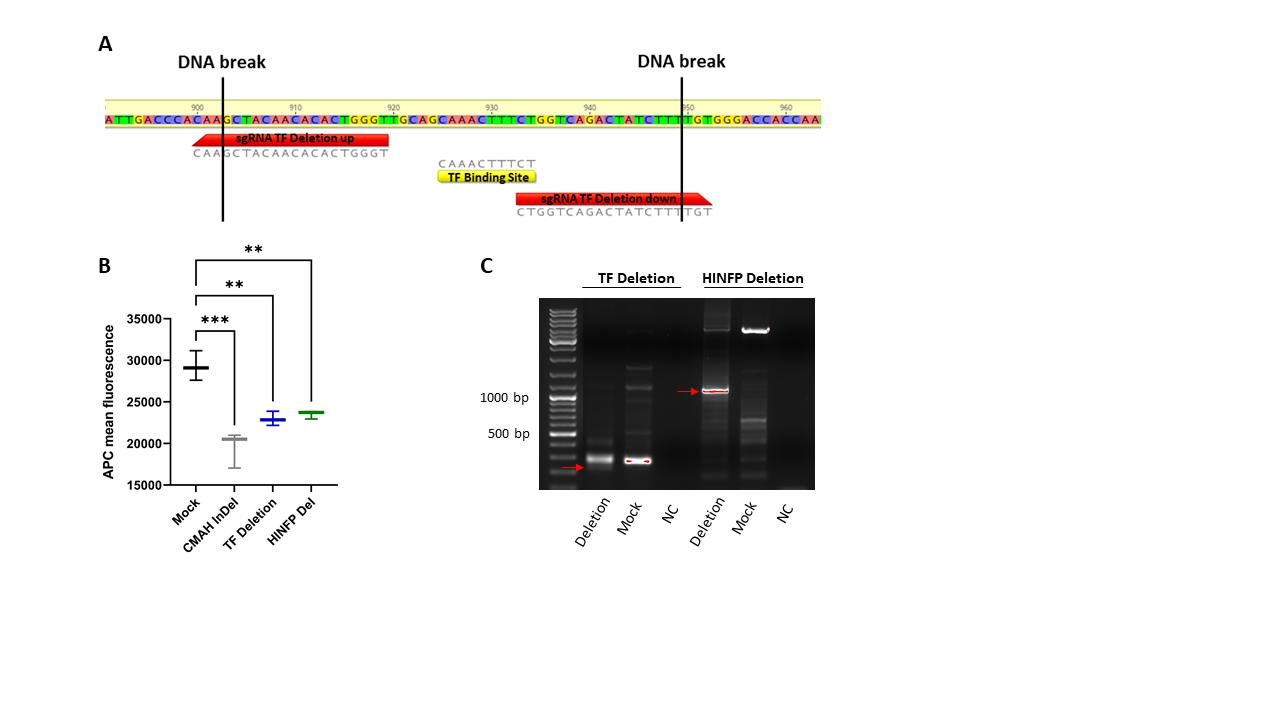

Supplement: Supplementary file 2 — Supporting information. [file BIT-119-832-s001.png]
