# Supplementary material for: Loss of a newly discovered microRNA in Chinese hamster ovary cells leads to upregulation of N‐glycolylneuraminic acid sialylation on monoclonal antibodies
Source: Biotechnol Bioeng. 2022 Jan 14;119(3):832–44. doi: 10.1002/bit.28015 (PMC9306616; doi:10.1002/bit.28015)
Supplement: Supplementary file 3 — Supporting information. [file BIT-119-832-s004.docx]

**Supplementary Table S1**: Utilized sgRNAs for targeted genome editing of Clone B by Cas9.

| **sgRNA** | **Sequence (5‘ 🡪 3‘)** |
| --- | --- |
| CMAH InDel | CTGCTTTTGCCCGAGGATGG |
| TF Deletion up | ACCCAGTGTGTTGTAGCTTG |
| TF Deletion down | CTGGTCAGACTATCTTTTGT |
| HINFP Deletion up | TTACCCTAACGTGTTCTTCA |
| HINFP Deletion down | CGAAAACGAGGGTGTCCTGA |

**Supplementary Table S2:** Primers utilized for deletion PCR of Cas9 edited CHO cells.

| **Primer** | **Sequence (5‘ 🡪 3‘)** | **PCR product size**  **WT** | **PCR product size Deletion** |
| --- | --- | --- | --- |
| TF Deletion Fw | CATGCTAACAAGGCAACCACC | 290 bp | 250 bp |
| TF Deletion Rev | CTGAGCCATGTGGCAGAATA |  |  |
| HINFP Deletion Fw | ACCACTTGCTTGCTAACCCA | - | 1104 bp |
| HINFP Deletion Rev | TGGCCAACAGGGAAACAAGA |  |  |
