# Supplementary material for: Loss of a newly discovered microRNA in Chinese hamster ovary cells leads to upregulation of N‐glycolylneuraminic acid sialylation on monoclonal antibodies
Source: Biotechnol Bioeng. 2022 Jan 14;119(3):832–44. doi: 10.1002/bit.28015 (PMC9306616; doi:10.1002/bit.28015)
Supplement: Supplementary file 4 — Supporting information. [file BIT-119-832-s003.docx]

**Supplementary Figure S1:** Normalized expression of mature miR-111 in Clone A and Clone B at different cultivation days, as determined by qRT-PCR.

**Supplementary Figure S2:** (A) Overview on genomic loci of the guide RNA pair (red) designed to cut out the predicted HINF-P transcription factor binding site (yellow) in Clone B. Predicted DNA break points are indicated. (B) Flow cytometry mediated analysis of NGNA sialylation of surface glycoproteins after CRISPR/Cas9-mediated knockout of either the putative HINF-P TF binding site (TF Deletion) or the HINF-P gene (HINF-P Del). CMAH knockout (CMAH In Del) served as positive control. Data are presented as APC mean fluorescence ± SD of N=3 analyses. Statistical analysis was performed against negative control cells (** p<0.01; *** p<0.001). (C) Deletion PCR results of gRNA pairs used to induce HINF-P TF binding site (TF Deletion) or HINF-P gene (HINFP Deletion) deletion. Cells were harvested 3 days post transfection for DNA extraction and subsequent deletion PCR. Expected deletion PCR products are indicated by red arrows.
